# Facial morphometric differences across face databases: influence of ethnicities and sex

**DOI:** 10.3389/fnins.2023.1130867

**Published:** 2023-06-19

**Authors:** Luis Carlos Pereira Monteiro, Rachel Coelho Ripardo, Nelson Torro-Alves, Givago Silva Souza

**Affiliations:** ^1^Neuroscience and Cell Biology Graduate Program, Institute of Biological Sciences, Federal University of Pará, Belém, Brazil; ^2^Center for Tropical Medicine, Federal University of Pará, Belém, Brazil; ^3^Neuroscience and Behavior Graduate Program, Center for Behavioral Theory and Research, Federal University of Pará, Belém, Brazil; ^4^Department of Psychology, Federal University of Paraíba, João Pessoa, Brazil

**Keywords:** morphometric geometrics, facial asymmetry, face databases, ethnicity, admixed population

## Abstract

The scientific need for standardized, high-quality facial stimuli has driven the creation of several face image databases in recent years. These stimuli are particularly important in facial asymmetry research. However, previous studies have reported facial anthropometric differences across a variety of ethnicities. This highlights the need to investigate whether these differences can also impact the use of face image databases, particularly in facial asymmetry research. In this study, we investigated facial asymmetry-based morphometric differences between the multi-ethnic Chicago Face Database (CFD) and the LACOP Face Database, which is composed of Brazilian subjects. We found reliable differences in facial asymmetry between the two databases, which were related to ethnic groups. Specifically, differences in eye and mouth asymmetry seem to drive these differences. The asymmetry-based morphometric differences among databases and ethnicities found in this study reinforce the necessity of creating multi-ethnic face databases.

## 1. Introduction

Given its biological and social importance, the face holds an important position across a variety of disciplines. The face offers several socially important information, such as individual identities (e.g., sex, age group, ethnicity), non-verbal language (e.g., social attention and emotional state), health, beauty, and attractiveness (Freiwald et al., 2016). The number of studies involving faces as stimuli has increased in recent years, highlighting the need for researchers to access publicly available sets of high-quality standardized face images (Workman and Chatterjee, 2021).

Several face image databases have been created in recent years across different countries and for various purposes (e.g., Phillips et al., 1998; Ebner et al., 2010; Ma et al., 2015; Chen et al., 2021). An example of a validated and widely used face database is the Chicago Face Database (CFD, Ma et al., 2015), which has been applied in various fields, including experimental psychology and computer vision (for recent examples see, respectively, Dupree et al., 2021 and Higgins et al., 2021). In Brazil, the LACOP Face Database was developed for similar purposes, but with Brazilian participants (Aznar-Casanova et al., 2014). The LACOP Face Database includes faces showing various expressions (happiness, sadness, fear, anger, surprise, disgust, and the neutral face) and views of the face (frontal and 45° tilted), with application in experimental psychology and developmental studies (e.g., Aguiar et al., 2017).

These face databases enable researchers to investigate how facial features can influence our perceptions of other individuals (Workman and Chatterjee, 2021). One frequently studied facial feature in face perception research is facial asymmetry (Little, 2014). Facial asymmetry has been proposed to reflect genetic factors and environmental stressors during human development (Palmer and Strobeck, 1986; Livshits and Kobyliansky, 1991). Consequently, facial asymmetry has been used as an indicator of ontogenetic (e.g., diseases, malnutrition, toxins) and genetic instability, and is important in both clinical (e.g., orthodontics) and social (e.g., aesthetic perception of faces) contexts (Rhodes, 2006; Thiesen et al., 2015).

Some studies have investigated the aesthetic perception of faces using CFD in non-US populations. For example, in populations from Germany (Klümper et al., 2020; Mertens et al., 2021), Italy (Balconi et al., 2020), Ireland (Ho and Newell, 2020), Australia (Ecker and Rodricks, 2020; White et al., 2021), and specifically in studies investigating the aesthetics perception of faces and its relationship with facial asymmetry, such as in a population from the Netherlands (Roth et al., 2022), England (Aksentijevic et al., 2021), Brazil (Monteiro et al., 2022), or those that do not report where the participants are from Lee et al. (2021) and Staller and Randler (2021).

As facial features, including facial asymmetry, are influenced by genetic and population-specific factors (Richmond et al., 2018; Rolfe et al., 2018), it is reasonable to assume that facial asymmetry values may vary between face databases composed of geographically specific populations. Furthermore, face perception can be influenced by the ethnicity of the face presented, particularly when it is different from that of the observer (Rhodes et al., 2005; Dudarev et al., 2022). Therefore, it is possible to question to what extent the facial asymmetry from different face databases is suitable to be presented to a given population when the proposed experimental design does not account the ethnicity of the face or the observer. The first step in addressing this question is to investigate the consistency of asymmetry measures across different face databases.

Previous studies have compared morphometric facial features among individuals from various ethnic groups. Sajid et al. (2018) reported differences in facial asymmetry among monoracial groups, including African, Asian, Hispanic, and European ethnicities. Specifically, differences in facial asymmetry were found in the lower parts of the face when comparing databases. However, no investigation has focused on comparing morphometric facial features among groups of individuals with mixed ethnic origins. The present study aims to compare the morphometric differences in facial asymmetry between two facial databases: the widely used CFD and the LACOP face database. CFD was selected because it contains faces from both monoracial (CFD Ma et al., 2015) and multiracial (CFD-MR, Ma et al., 2021) individuals, while LACOP was chosen due to its composition of Brazilian individuals, a population with a prevalent mixed ethnic origin. Therefore, this study aims to explore differences not only among monoracial groups but also among multiracial groups, such as the Brazilian LACOP database and the multiracial set of the Chicago Face Database.

## 2. Materials and methods

### 2.1. Face image databases

**Figure 1:**
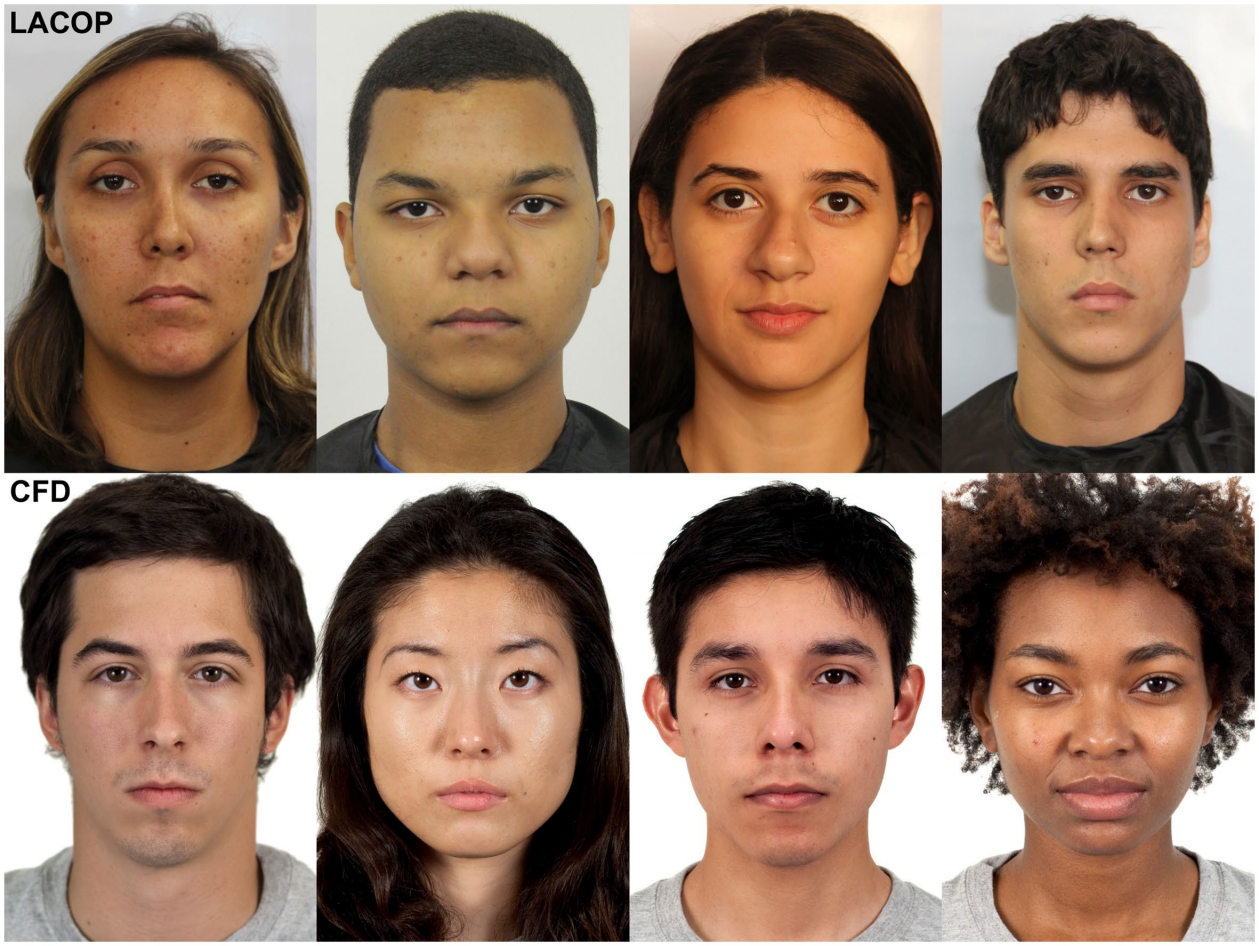
Examples of faces selected in the LACOP Face Database (Aznar-Casanova et al., 2014) and Chicago Face Database (CFD, Ma et al., 2015, 2021).

We selected 179 faces from two image databases: LACOP face database (Aznar-Casanova et al., 2014) and the Chicago Face Database (CFD, Ma et al., 2015, 2021) version 3.0. LACOP has 58 images of neutral faces in the open mouth and closed mouth variation, of which only the closed mouth variation was selected (*N* = 29, 13 females, 16 males). Regarding ethnicity, all faces in LACOP database were from a Brazilian admixed population. The main CFD set, available at,^1^ has 597 photographs of faces that vary in four different ethnicities—Asian, Black (African Americans), Latin, and White—of which we selected 30 faces for each ethnicity (*N* = 120). Multiracial expansion of CFD (CFD-R, Ma et al., 2021) is composed by 88 photographs of faces of which we selected 30 faces. Each selected group had 15 photos of female faces and 15 photos of male faces with neutral expression. Both databases have high-resolution photographs. The selection was randomized within each ethnicity. Examples of selected faces are shown in Figure 1.

### 2.2. Asymmetry measurement

**Figure 2:**
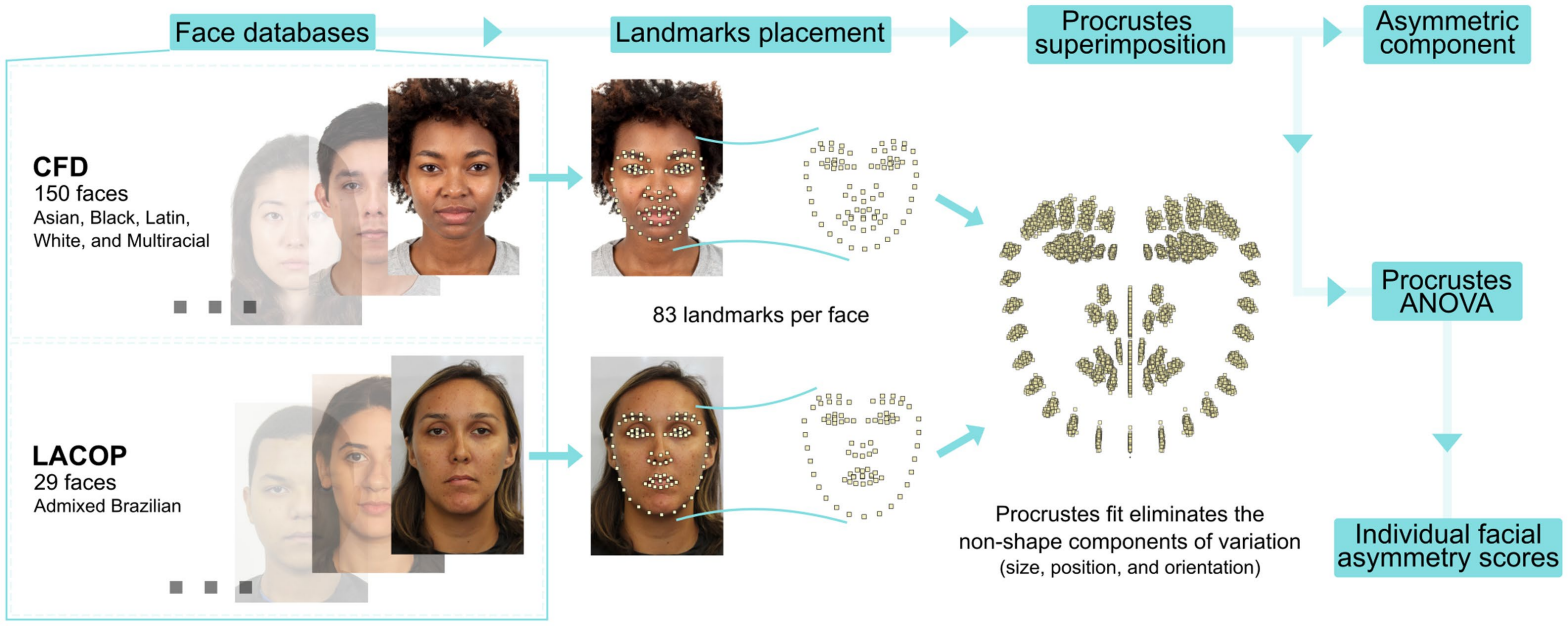
Morphometric geometrics procedure. Eighty-three landmarks were positioned on selected faces from two face databases, the LACOP Face Database (Aznar-Casanova et al., 2014) and the Chicago Face Database (CFD, Ma et al., 2015, 2021). A Procrustes superimposition was used to eliminate non-shape components of variation. Standardized data was used to obtain the asymmetric component of facial shape. In addition, Procrustes ANOVA was performed to get individual facial asymmetry scores for each face.

Eighty-three facial landmarks were included in the selected faces using Face++ API (Megvii Technology)^2^ to measure facial asymmetry. Face++ API has proven to be reliable for correctly marking landmarks in other studies (Sajid et al., 2018; Küntzler et al., 2021). Facial asymmetry was then calculated following geometric morphometric techniques as described in Klingenberg (2015) (Figure 2). In the MorphoJ software (Klingenberg, 2011), a Procrustes superimposition for shape with object symmetry was used to standardize landmark configuration in relation to shape scale, rotation, and translation. Object symmetry assumes that the left and right halves of the faces are mirror images of each other. The asymmetric component (i.e., the difference between original and mirrored landmark configuration in relation to the mean shape within faces) was then estimated from the Procrustes superimposition. Furthermore, individual facial asymmetry scores were obtained through a Procrustes ANOVA, a mixed two-away ANOVA model that includes the individual, the side of the face and their interaction as factors. Facial asymmetry scores correspond to mean squares value of the individual and side interaction corrected for error variance in the Procrustes ANOVA, and were calculated using Mahalanobis distances (scaled to the sample asymmetry variation)—which are used to remove the effect of anisotropic variation that can bias estimates based on classical Procrustes distances (Klingenberg and Monteiro, 2005). The procedure for calculating asymmetry was also applied to the landmarks of the following facial features separately: eyes (including eyebrows), nose, mouth, and contour. These facial features were chosen because (i) they are commonly used in human face research (e.g., Fang et al., 2011; Diego-Mas et al., 2020) and (ii) they are easily identifiable through the landmarks we utilized.

### 2.3. Data analysis

The asymmetric component of facial shape was examined using Canonical Variate Analysis (CVA). CVA was performed to test database, sex, and ethnic group differences. A two-way ANOVA was used to verify the effect of sex and ethnicity on facial asymmetry scores. The visual inspection of the model’s residuals in association with Shapiro–Wilk and Bartlett test was used to verify, respectively, if the assumptions of normality and homogeneity of variances were met. No deviation was identified. A bootstrapped Welch two sample *t*-test with 1,000 replications was used to verify whether the facial asymmetry scores varied according to the face database. Morphometrics and CVA analysis were performed using MorphoJ version 1.07a (Klingenberg, 2011). Other tests and graphical representations were performed using R version 4.0.4 (R Core Team, 2019) and the following packages: rstatix (Kassambara, 2020), MKinfer (Kohl, 2020), performance (Lüdecke et al., 2021), ggplot2 (Wickham, 2016), and ggridges (Wilke, 2021).

## 3. Results

### 3.1. Face

**Figure 3:**
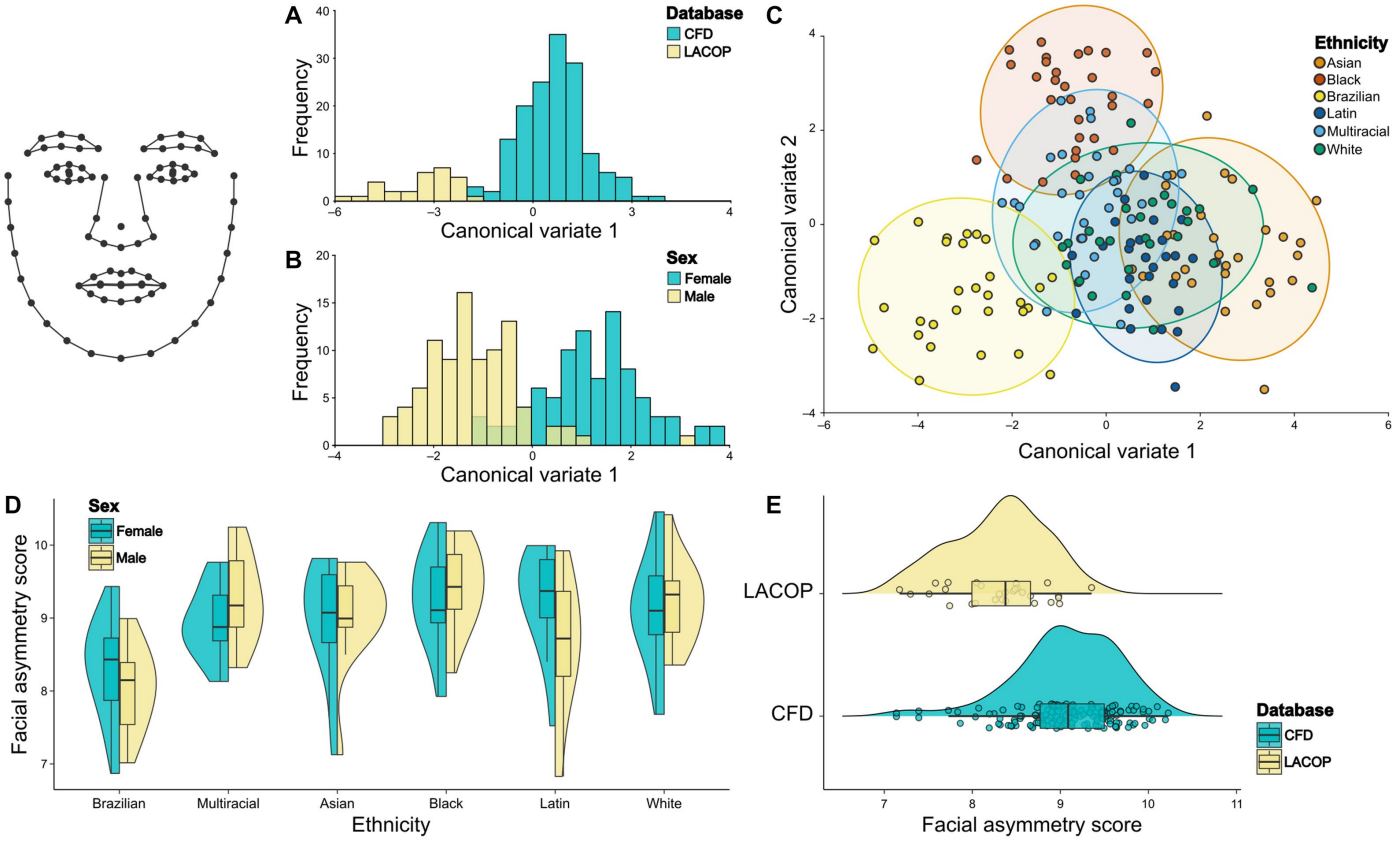
Face shape analysis. Canonical Variate Analysis (CVA) of asymmetric component of facial shape using database **(A)**, sex **(B)**, and ethnicity **(C)**. Individual facial asymmetry scores as a function of sex and ethnicity **(D)** and database **(E)**. In **(C)**, 95% confidence ellipses for each group are shown. In **(D,E)**, split violin plots show a kernel density estimate of the distributions. Dots represent single data points. The boxplots represent the median (midline), upper and lower quartiles (box), and data range (whiskers). The wireframe in the upper left corner illustrates the face shape and landmarks used. CFD, Chicago Face Database; LACOP, LACOP Face Database.

Through CVA, we found significant differences in face mean shape regarding database (Figure 3A) and sex (Figure 3B). The Mahalanobis distance between the CFD and LACOP databases was 4.02 (*p* < 0.001). Regarding sex, the Mahalanobis distance between males and females was 2.38 (*p* < 0.001). We also found a significant difference in face mean shape regarding the ethnicity with visual overlap between the groups (Figure 3C). Mahalanobis distances among ethnic groups are shown in Table 1.

**Table 1:** Pairwise Mahalanobis distance matrix between ethnic groups.

Face	Asian	Black	Brazilian	Latin	Multiracial
Black	4.85 (<0.001)				
Brazilian	5.66 (<0.001)	4.80 (<0.001)			
Latin	4.04 (<0.001)	4.43 (<0.001)	4.80 (<0.001)		
Multiracial	4.22 (<0.001)	3.62 (<0.001)	4.16 (<0.001)	3.82 (<0.001)	
White	3.69 (<0.001)	4.03 (<0.001)	4.57 (<0.001)	3.40 (<0.001)	3.24 (<0.001)
Eyes	Asian	Black	Brazilian	Latin	Multiracial
Black	2.26 (<0.001)				
Brazilian	2.28 (<0.001)	2.35 (<0.001)			
Latin	1.84 (0.03)	2.58 (<0.001)	2.58 (<0.001)		
Multiracial	1.66 (0.24)	2.22 (<0.001)	2.00 (0.001)	2.36 (<0.001)	
White	1.53 (0.58)	2.23 (<0.001)	2.21 (<0.001)	2.11 (0.001)	1.45 (0.82)
Nose	Asian	Black	Brazilian	Latin	Multiracial
Black	0.97 (0.01)				
Brazilian	0.72 (0.21)	1.07 (0.003)			
Latin	0.58 (0.50)	1.03 (0.01)	1.06 (0.005)		
Multiracial	0.60 (0.53)	0.77 (0.18)	0.87 (0.09)	0.67 (0.40)	
White	0.31 (0.97)	1.05 (0.01)	0.46 (0.80)	0.77 (0.25)	0.70 (0.41)
Mouth	Asian	Black	Brazilian	Latin	Multiracial
Black	0.76 (0.95)				
Brazilian	1.44 (0.003)	1.32 (0.02)			
Latin	1.23 (0.17)	1.15 (0.25)	1.15 (0.11)		
Multiracial	1.06 (0.51)	1.19 (0.23)	1.20 (0.08)	1.01 (0.56)	
White	1.19 (0.26)	1.12 (0.34)	1.82 (<0.001)	1.35 (0.05)	1.19 (0.25)
Contour	Asian	Black	Brazilian	Latin	Multiracial
Black	1.57 (0.007)				
Brazilian	2.27 (<0.001)	1.79 (<0.001)			
Latin	2.13 (<0.001)	1.80 (<0.001)	1.75 (<0.001)		
Multiracial	1.64 (0.002)	1.26 (0.24)	1.68 (<0.001)	1.20 (0.22)	
White	1.79 (<0.001)	1.44 (0.03)	1.43 (0.02)	1.38 (0.02)	1.26 (0.15)

The *p*-values from permutation tests (10000) are given in brackets.

From the results of the two-way ANOVA, we found an effect of ethnicity [*F*_(17.3,5)_ = 10.5, *p* < 0.001], but not of sex [*F*_(0,1)_ = 0.13, *p* = 0.72] or the interaction between sex and ethnicity [*F*_(3.3,5)_ = 2.01, *p* = 0.081] on the individual facial asymmetry scores. Tukey HSD posthoc test found significant differences in individual facial asymmetry scores between Brazilian and Asian (*p* < 0.001), Brazilian and Black (*p* < 0.001), Brazilian and Latin (*p* < 0.001), Brazilian and White (*p* < 0.001), and Brazilian and Multiracial (*p* < 0.001) ethnic groups. No significant differences were found between the other ethnic groups. The distribution of the facial asymmetry scores by ethnic groups and sex is shown in Figure 3D. In addition, a bootstrapped welch two sample *t*-test showed that the means of LACOP (*M* = 8.29) and CFD (*M* = 9.08) individual facial asymmetry scores differ significantly [*t*_(41.7)_ = 6.97, 95% bootstrap CI (0.57, 1.01), *p* < 0.001, bootstrapped *p* < 0.001]. The distribution of the scores by database is shown in Figure 3E.

Once we found significant differences between the face databases, we reanalyzed the data using subsets of landmarks for the following parts of the face: eyes (including eyebrows), nose, mouth and contour as follows.

### 3.2. Eyes

**Figure 4:**
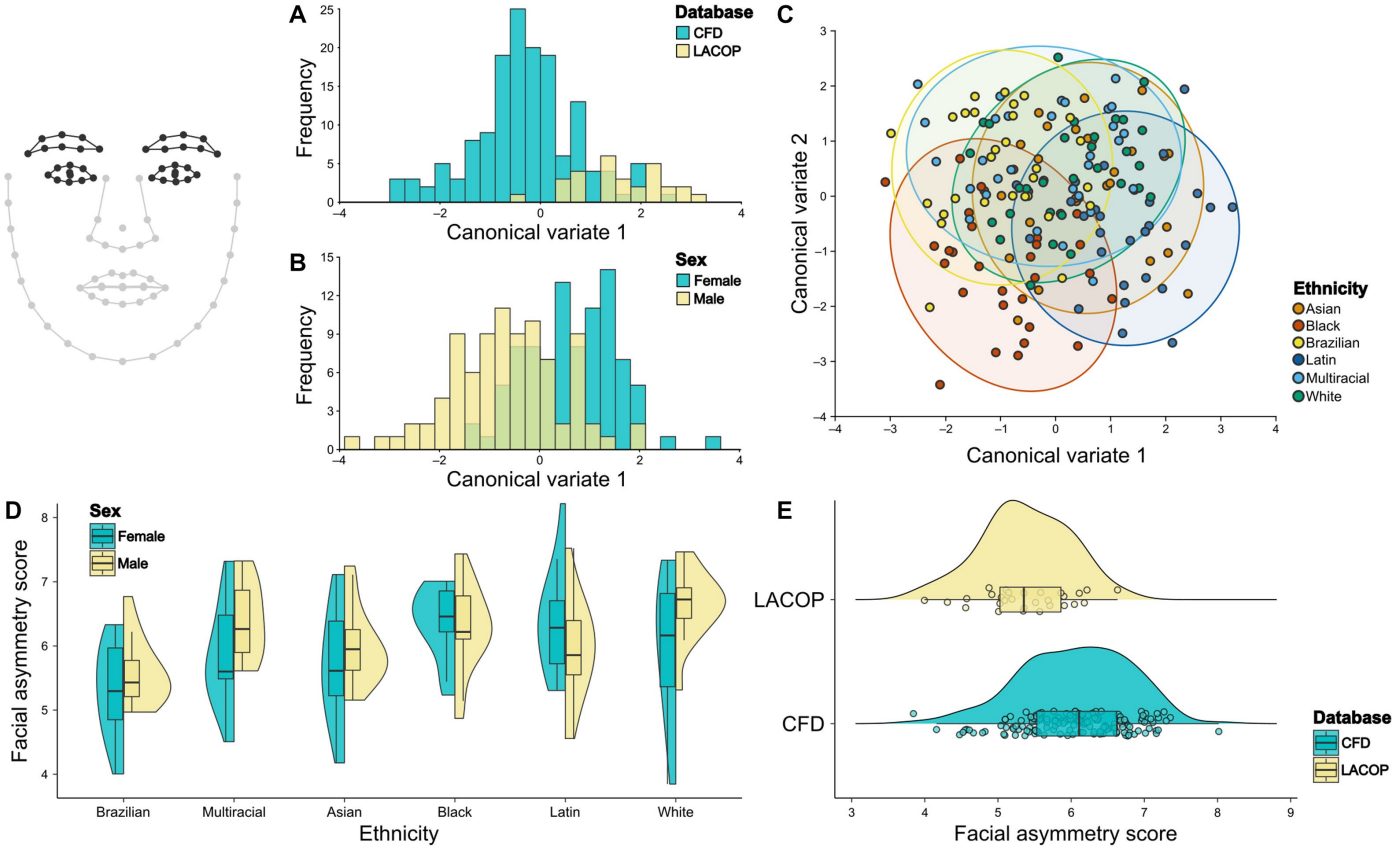
Eyes shape analysis. Canonical Variate Analysis (CVA) of asymmetric component of eyes shape using database **(A)**, sex **(B)**, and ethnicity **(C)**. Individual facial asymmetry scores as a function of sex and ethnicity **(D)** and database **(E)**. In **(C)**, 95% confidence ellipses for each group are shown. In **(D,E)**, split violin plots show a kernel density estimate of the distributions. Dots represent single data points. The boxplots represent the median (midline), upper and lower quartiles (box), and data range (whiskers). The wireframe in the upper left corner illustrates the face shape and landmarks used. Eyes and eyebrows landmarks are highlighted. CFD, Chicago Face Database; LACOP, LACOP Face Database.

We found significant differences in eyes mean shape regarding database (Figure 4A) and sex (Figure 4B) through CVA analysis. The Mahalanobis distance between the CFD and LACOP databases was 1.89 (*p* < 0.001). Regarding sex, the Mahalanobis distance between males and females was 1.31 (*p* < 0.001). We also found significant differences in eye mean shape between all ethnicities, except between Asian and White, Asian and Multiracial, and Multiracial and White ethnic groups (Figure 4C; Table 1).

From the results of the two-way ANOVA, we found an effect of ethnicity [*F*_(15.5,5)_ = 5.93, *p* < 0.001], but not of sex [*F*_(1.7,1)_ = 3.49, *p* = 0.06] or the interaction between sex and ethnicity [*F*_(5.1,5)_ = 2.07, *p* = 0.07] on the individual facial asymmetry scores using the eyes and eyebrows landmarks. Tukey HSD posthoc test found significant differences in individual facial asymmetry scores between Brazilian and Black (*p* < 0.001), Brazilian and Latin (*p* = 0.01), Brazilian and White (*p* < 0.001), and Brazilian and Multiracial (*p* = 0.007) ethnic groups. No significant differences were found between the other ethnic groups. The distributions of the facial asymmetry scores using eyes and eyebrows landmarks by ethnic groups and sex are shown in Figure 4D. In addition, a bootstrapped welch two sample *t*-test showed that the means of LACOP (*M* = 5.37) and CFD (*M* = 6.04) individual facial asymmetry scores based on eyes landmarks differ significantly [*t*_(45.4)_ = 5.28, 95% bootstrap CI (0.43, 0.94), *p* < 0.001, bootstrapped *p* < 0.001]. The distribution of the scores by database is shown in Figure 4E.

### 3.3. Nose

**Figure 5:**
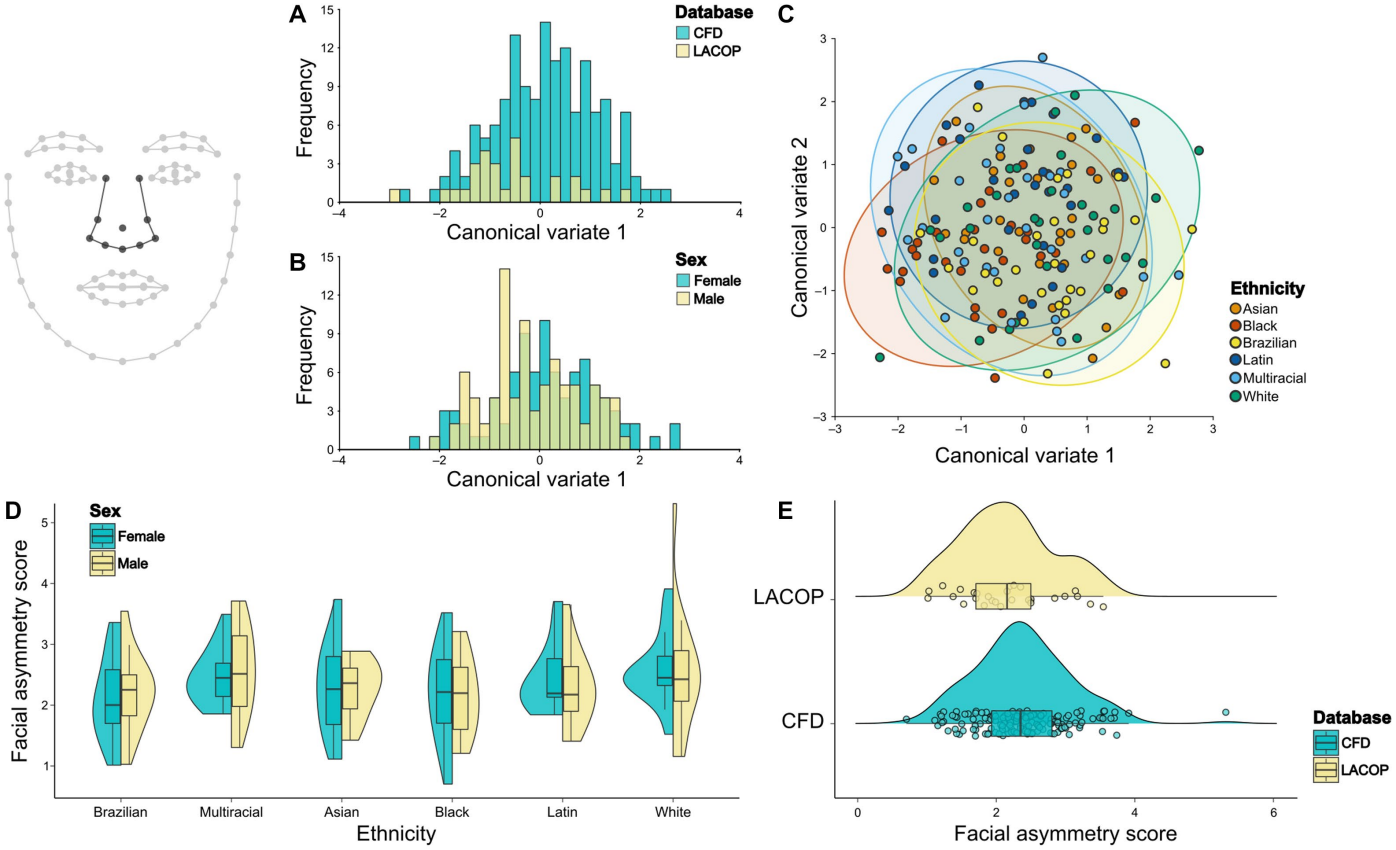
Nose shape analysis. Canonical Variate Analysis (CVA) of asymmetric component of eyes shape using database **(A)**, sex **(B)**, and ethnicity **(C)**. Individual facial asymmetry scores as a function of sex and ethnicity **(D)** and database **(E)**. In **(C)**, 95% confidence ellipses for each group are shown. In **(D,E)**, split violin plots show a kernel density estimate of the distributions. Dots represent single data points. The boxplots represent the median (midline), upper and lower quartiles (box), and data range (whiskers). The wireframe in the upper left corner illustrates the face shape and landmarks used. Nose landmarks are highlighted. CFD, Chicago Face Database; LACOP, LACOP Face Database.

We found no significant difference in nose mean shape regarding database (Figure 5A) or sex (Figure 5B) through CVA analysis. The Mahalanobis distance between the CFD and LACOP databases was 0.71 (*p* = 0.06). Regarding sex, the Mahalanobis distance between males and females was 0.28 (*p* = 0.74). On the other hand, we found significant differences in nose mean shape between Black and Asian, Black and Latin, Black and White, Black and Brazilian, and Brazilian and Latin ethnic groups (Figure 5C; Table 1).

From the results of the two-way ANOVA, we found no effect of ethnicity [*F*_(4.48,5)_ = 1.89, *p* = 0.09], sex [*F*_(0.01,1)_ = 0.11, *p* = 0.91] or the interaction between sex and ethnicity [*F*_(0.40,5)_ = 0.17, *p* = 0.97] on the individual facial asymmetry scores using the nose landmarks. The distributions of the facial asymmetry scores using nose landmarks by ethnic groups and sex are shown in Figure 5D. A bootstrapped welch two sample *t*-test showed that the means of LACOP (*M* = 2.16) and CFD (*M* = 2.38) individual facial asymmetry scores based on nose landmarks do not differ significantly (*t*_(39.9)_ = 1.59, 95% bootstrap CI [−0.05, 0.47], *p* = 0.12, bootstrapped *p* = 0.11). The distribution of the scores by database is shown in Figure 5E.

### 3.4. Mouth

**Figure 6:**
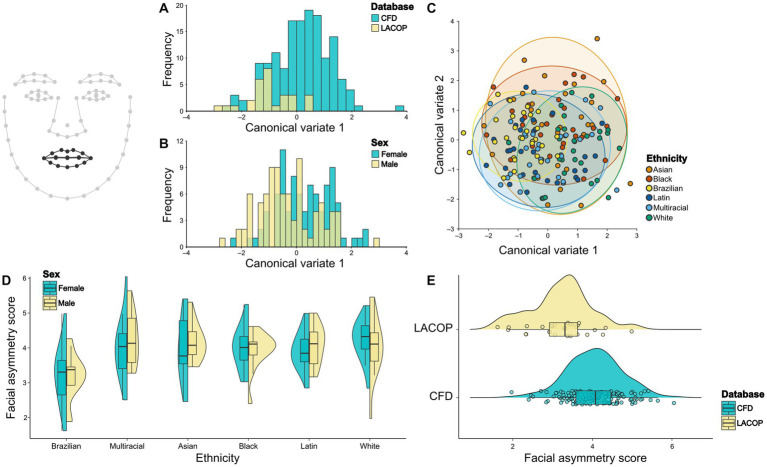
Mouth shape analysis. Canonical Variate Analysis (CVA) of asymmetric component of eyes shape using database **(A)**, sex **(B)**, and ethnicity **(C)**. Individual facial asymmetry scores as a function of sex and ethnicity **(D)** and database **(E)**. In **(C)**, 95% confidence ellipses for each group are shown. In **(D,E)**, split violin plots show a kernel density estimate of the distributions. Dots represent single data points. The boxplots represent the median (midline), upper and lower quartiles (box), and data range (whiskers). The wireframe in the upper left corner illustrates the face shape and landmarks used. Mouth landmarks are highlighted. CFD, Chicago Face Database; LACOP, LACOP Face Database.

Through CVA, we found significant differences in mouth mean shape regarding database (Figure 6A), but not sex (Figure 6B). The Mahalanobis distance between the CFD and LACOP databases was 1.19 (*p* = 0.003). Regarding sex, the Mahalanobis distance between males and females was 0.61 (*p* = 0.42). We also found significant differences in mouth mean shape between Brazilian and Asian, Brazilian and Black, and Brazilian and White ethnic groups (Figure 6C; Table 1).

From the results of the two-way ANOVA, we found an effect of ethnicity [*F*_(18.3,5)_ = 7.24, *p* < 0.001], but not sex [*F*_(0.11,1)_ = 0.22, *p* = 0.64] or the interaction between sex and ethnicity [*F*_(1.12,5)_ = 0.44, *p* = 0.82] on the individual facial asymmetry scores using the mouth landmarks. The distributions of the facial asymmetry scores using mouth landmarks by ethnic groups and sex are shown in Figure 6D. Tukey HSD posthoc test found significant differences in individual facial asymmetry scores between Brazilian and Asian (*p* < 0.001), Brazilian and Black (*p* = 0.001), Brazilian and Latin (*p* < 0.001), Brazilian and White (*p* < 0.001), and Brazilian and Multiracial (*p* < 0.001) ethnic groups. No significant differences were found between the other ethnic groups. In addition, a bootstrapped welch two sample *t*-test showed that the means of LACOP (*M* = 3.20) and CFD (*M* = 4.05) individual facial asymmetry scores differ significantly [*t*_(37.2)_ = 5.56, 95% bootstrap CI (0.55, 1.14), *p* < 0.001, bootstrapped *p* < 0.001]. The distribution of the scores by database is shown in Figure 6E.

### 3.5. Contour

**Figure 7:**
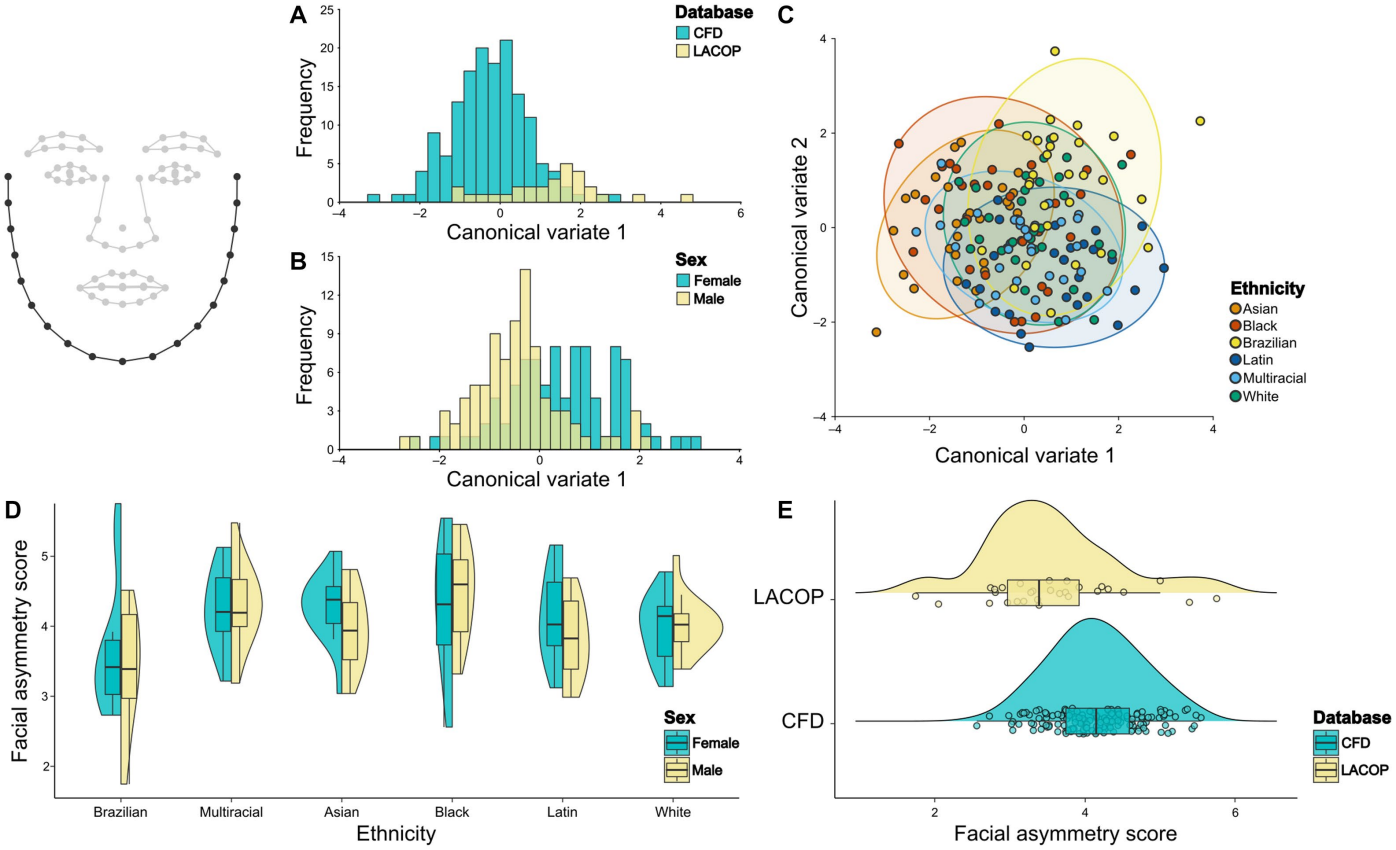
Face contour shape analysis. Canonical Variate Analysis (CVA) of asymmetric component of eyes shape using database **(A)**, sex **(B)**, and ethnicity **(C)**. Individual facial asymmetry scores as a function of sex and ethnicity **(D)** and database **(E)**. In **(C)**, 95% confidence ellipses for each group are shown. In **(D,E)**, split violin plots show a kernel density estimate of the distributions. Dots represent single data points. The boxplots represent the median (midline), upper and lower quartiles (box), and data range (whiskers). The wireframe in the upper left corner illustrates the face shape and landmarks used. Face contour landmarks are highlighted. CFD, Chicago Face Database; LACOP, LACOP Face Database.

Through CVA, we found significant differences in face contour mean shape regarding database (Figure 7A) and sex (Figure 7B). The Mahalanobis distance between the CFD and LACOP databases was 1.49 (*p* < 0.001). Regarding sex, the Mahalanobis distance between males and females was 0.89 (*p* = 0.003). We also found significant differences in eye mean shape between ethnicities, except between Multiracial and Asian, Multiracial and Latin, and Multiracial and White ethnic groups (Figure 7C). Mahalanobis distances among ethnic groups are shown in Table 1.

From the results of the two-way ANOVA, we found an effect of ethnicity [*F*_(13.1,5)_ = 6.06, *p* < 0.001], but not sex [*F*_(0.41,1)_ = 0.94, *p* = 0.33] or the interaction between sex and ethnicity [*F*_(2.05,5)_ = 0.94, *p* = 0.45] on the individual facial asymmetry scores using the face contour landmarks. The distributions of the facial asymmetry scores using contour landmarks by ethnic groups and sex are shown in Figure 7D. Tukey HSD posthoc test found significant differences in individual facial asymmetry scores between Brazilian and Asian (*p* = 0.01), Brazilian and Black (*p* < 0.001), and Brazilian and Multiracial ethnic groups (*p* < 0.001). No significant differences were found between the other ethnic groups. In addition, a bootstrapped welch two sample *t*-test showed that the means of LACOP (*M* = 3.54) and CFD (*M* = 4.15) individual facial asymmetry scores differ significantly [*t*_(33.3)_ = 3.49, 95% bootstrap CI (0.28, 0.94), *p* = 0.001, bootstrapped *p* = 0.006]. The distribution of the scores by database is shown in Figure 7E.

## 4. Discussion

In this work, we investigated differences in facial shape, specifically focusing on facial asymmetry, between two face databases using geometric morphometric analyses. Our findings indicate that both the face database and the ethnicity of the faces had a significant impact on morphometric differences related to facial asymmetry, even for geographically distinct multiracial groups. Specifically, consistent differences between the databases were observed in the eyes and mouth when analyzing the face landmarks data separately for each part of the face (eyes, nose, mouth, and face contour).

Face databases are a valuable tool in research involving populations from diverse countries and culture (Workman and Chatterjee, 2021). In addition, several facial anthropometric differences among ethnic groups have been reported (e.g., Zhuang et al., 2010; Fang et al., 2011; Zacharopoulos et al., 2016). Our findings suggest that facial asymmetry measurements may not be fully comparable across different population, which could potentially affect the reliability of research results carried out with different databases.

Sajid et al. (2018) previously reported differences in facial asymmetry between African, Asian, Hispanic, and European ethnic groups. Our study extends these differences to the admixed Brazilian population, as the LACOP (composed of Brazilian subjects) differed significantly from the CFD (composed of Asian, Black, Latin, and White subjects). We also included the CFD multiracial extension which also showed consistent differences. These findings suggest that even geographically distinct multiracial populations may exhibit morphometric differences regarding facial asymmetry, which could be due to genetic and environmental divergences resulting from geographical variation (Richmond et al., 2018; Zhang et al., 2022). Our results are also congruent with previous studies that have highlighted differences in eye and mouth facial dimensions across different populations (Fang et al., 2011).

Sajid et al. (2018) also found differences related to sex. Although we found significant sex differences regarding the face mean shape, we did not find differences related to facial asymmetry scores. Inconsistencies in sex differences may be related to sample size within each ethnic group (~15 males to 15 females). Moreover, studies on sex differences in facial asymmetry often report mixed results (Smith, 2000; Hardie et al., 2005; Primozic et al., 2012; Bugaighis et al., 2013; Sajid et al., 2018). Furthermore, it is important to highlight methodological similarities and differences between the present study and Sajid et al. (2018). In Sajid et al. (2018), facial landmarks were added using an automatic insertion via Face++ API, followed by face alignment using a supervised descent method to calculate Euclidean distances between landmarks and pupils. Facial asymmetry was calculated by taking the difference between the same distance from both facial sides. In contrast, in the present study, we also used Face++ API for automatic facial landmark detection, but we employed geometric morphometric methods (Procrustes superimposition and Procrustes ANOVA) to calculate facial asymmetry. This approach has the advantage of providing more reliable estimates of facial asymmetry (Klingenberg, 2015).

Although the LACOP database differed from the other database in terms of facial asymmetry, the Brazilian face database may have the bias of being composed of faces predominantly from Northeast Brazil. This highlights the necessity of expand the variability within the LACOP database by incorporating faces from other regions of Brazil, as the population can be quite heterogeneous in terms of facial features, due to mixed ancestry (Pena et al., 2020).

Given the labor-intensive process of measuring asymmetry across a large number of facial photographs, we made a deliberate choice to select only two databases based on their quality and ethnic diversity. Our selection criteria prioritized databases that included not only monoracial but also multiracial groups. It should be noted that the use of only two face databases may limit the generalizability of our findings to Brazilian and US populations, as represented in the databases we employed. Therefore, we recommend that future studies should compare results across multiple facial photograph databases, with representation from different geographically distinct ethnic groups, to determine the consistency of our findings.

Many face image databases used in scientific research have low ethnic representation, although this is changing (Chen et al., 2021; Ma et al., 2021). The population-based differences previously reported and the results of this study strengthen the idea of creating population-specific and multi-ethnic face databases, particularly in the use of facial asymmetry metrics. In addition, the results of this study may be important not only for face perception research but also for computer vision, since morphometric differences can be used to extract useful information from faces by automated means (Sajid et al., 2021), which can address real-life concerns and assist in the objective classification of ethnicities for affirmative action in different countries.

## Data availability statement

The original contributions presented in the study are included in the article/supplementary material, further inquiries can be directed to the corresponding author.

## Author contributions

LM and GS contributed to the conception and design of the study, performed the formal analysis, and wrote the first draft of the manuscript. NT-A provided the stimuli. LM collected and processed the data. NT-A and RR contributed to the writing and review of the final version of the manuscript. All authors contributed to the article and approved the submitted version.

## Funding

This work was supported by research grants: CAPES-PROCAD (CAPES-PROCAD #88887.200446/2018-00) and CNPq grant (431748/2016-0). LM received a CAPES scholarship for graduate students. GS and NT-A are CNPq Fellows. CNPq Productivity Grant to GS is 310845/2018-1. The funders had no role in the study design.

## Conflict of interest

The authors declare that the research was conducted in the absence of any commercial or financial relationships that could be construed as a potential conflict of interest.

## Publisher’s note

All claims expressed in this article are solely those of the authors and do not necessarily represent those of their affiliated organizations, or those of the publisher, the editors and the reviewers. Any product that may be evaluated in this article, or claim that may be made by its manufacturer, is not guaranteed or endorsed by the publisher.

## Footnotes

^1^ chicagofaces.org

^2^ faceplusplus.com
